# Identification of Biomarkers of Response to IFNg during Endotoxin Tolerance: Application to Septic Shock

**DOI:** 10.1371/journal.pone.0068218

**Published:** 2013-07-11

**Authors:** Florence Allantaz-Frager, Fanny Turrel-Davin, Fabienne Venet, Cécile Monnin, Amélie De Saint Jean, Véronique Barbalat, Elisabeth Cerrato, Alexandre Pachot, Alain Lepape, Guillaume Monneret

**Affiliations:** 1 Joint Unit Hospices Civils de Lyon-Biomérieux « sepsis », Hôpital Edouard Herriot, Lyon, France; 2 Cellular Immunology Laboratory, Hôpital Edouard Herriot, Lyon, France; 3 BioMérieux sa, Marcy l’Etoile, France; 4 Intensive Care Unit, Centre Hospitalier Lyon-Sud, Pierre-Bénite, France; University of Cincinnati, United States of America

## Abstract

The rapid development in septic patients of features of marked immunosuppression associated with increased risk of nosocomial infections and mortality represents the rational for the initiation of immune targeted treatments in sepsis. However, as there is no clinical sign of immune dysfunctions, the current challenge is to develop biomarkers that will help clinicians identify the patients that would benefit from immunotherapy and monitor its efficacy. Using an *in vitro* model of endotoxin tolerance (ET), a pivotal feature of sepsis-induced immunosuppression in monocytes, we identified using gene expression profiling by microarray a panel of transcripts associated with the development of ET which expression was restored after immunostimulation with interferon-gamma (IFN-γ). These results were confirmed by qRT-PCR. Importantly, this short-list of markers was further evaluated in patients. Of these transcripts, six (TNFAIP6, FCN1, CXCL10, GBP1, CXCL5 and PID1) were differentially expressed in septic patients’ blood compared to healthy blood upon *ex vivo* LPS stimulation and were restored by IFN-γ. In this study, by combining a microarray approach in an *in vitro* model and a validation in clinical samples, we identified a panel of six new transcripts that could be used for the identification of septic patients eligible for IFNg therapy. Along with the previously identified markers TNFa, IL10 and HLA-DRA, the potential value of these markers should now be evaluated in a larger cohort of patients. Upon favorable results, they could serve as stratification tools prior to immunostimulatory treatment and to monitor drug efficacy.

## Introduction

Sepsis is a major public health issue and remains the leading cause of death in the Intensive Care Units (ICU) with an estimated 6 million victims per year worldwide [1], [2]. Mortality remains high, ranging from 20% for sepsis to more than 50% for septic shock. More worrisome, a 75% increase in the incidence rate of severe sepsis has been observed over the past two decades, probably due to improved care of the elderly and to associated comorbidities in patients (cancer or diabetes for example) [1], [3].

The traditional view of sepsis has been greatly challenged within the past few years and it is now becoming evident that septic patients rapidly present with features of marked immunosuppression. Immunosuppression is believed to be responsible for the increased risk of nosocomial infections and mortality [2]. Reversal of sepsis-induced immune-paralysis could significantly reduce the occurrence of secondary infections and improve the prognosis of these patients [4], [5]. To this end, administration of immunostimulatory molecules might be a very effective therapy [6]. Indeed, interferon-gamma (IFN-γ) has been shown to be a promising drug to treat the anergy observed in monocytes from septic and trauma patients [7]–[11]. However, as there is no clinical sign of sepsis-induced immune dysfunctions that would help in predicting the risk of nosocomial occurrence, there is an urgent need to develop immunomonitoring tools that will allow the identification of patients that would benefit from immunostimulatory therapies and permit the monitoring of successful response to treatment.

Some features of sepsis-induced immune dysfunctions can be mimicked *in vitro* using healthy volunteers cells. In particular, the monocyte side of immunosuppression can be partly mimicked *in vitro* by an endotoxin tolerance model [12], [13]. Endotoxin tolerance (ET) is observed in cells that have been exposed to low concentration of endotoxin (e.g. Lipopolysaccharide, LPS) and that become transiently unresponsive to further challenge with endotoxin. Functionally, endotoxin-tolerant monocytes exhibit an increased phagocytic activity, impaired antigen presentation, decrease in secretion of pro-inflammatory cytokines and increase in secretion of anti-inflammatory cytokine [14]–[19].

In the current study, we took advantage of an *in vitro* model of ET to identify a panel of markers of response to IFNg treatment by gene expression profiling using microarrays. Importantly, this list of transcripts was validated in septic patients’ cells to select markers that were differentially expressed in patients compared to healthy donors and restored by IFN-γ. Using this bench to bedside approach, we identified a panel of 6 transcripts that now needs to be confirmed as monitoring markers in large multicentric studies.

## Materials and Methods

### PBMCs Isolation and ET Model

Citrated blood from healthy donors was obtained from EFS (Etablissement Français du Sang) and used immediately. According to EFS standardized procedures for blood donation, informed consent was obtained from healthy volunteers and personal data for blood donors were anonymized at time of blood donation and before blood transfer to our research lab. Peripheral blood mononuclear cells (PBMCs) were isolated from citrated venous blood by Ficoll-Paque density gradient centrifugation (Amersham Biosciences, Björkgatan, Sweden) and washed with PBS while the remaining red blood cells were lysed. Cells were cultured in 24-well plates at 2.10^6^ cells/ml in X-Vivo 20 Medium (Lonza, Verviers, Belgium). To induce the LPS-primed state, PBMCs were cultured in the presence or absence (control group) of 2 ng/ml LPS mix from *Escherichia coli* O55:B5, O127:B8 and O111:B4 (purified by gel filtration chromatography) (Sigma-Aldrich, Deisenhofen, Germany) [14] and incubated overnight at 37°C and 5% CO2 (15 hours). Following a washing step, PBMCs were incubated for an additional 24 hours in the presence or absence (control group stimulated or not with LPS) of rIFN-γ1b (Imukin, Boehringer, Ingelheim, Austria). Finally, cells were stimulated a second time by adding 100 ng/ml of LPS for another 6 hours [14]. For each condition, supernatants were recovered and stored at –80°C. Cells were harvested, lysed in RLT buffer and stored at –80°C until further processing.

### RNA Extraction

Total RNA was extracted from PBMCs using RNeasy Plus Mini kits (Qiagen, Hilden, Germany) or from whole blood using QIAamp RNA Blood Mini Kit (Qiagen). For each RNA extraction, the residual genomic DNA was digested using the gDNA Eliminator spin column (Qiagen). RNA was eluted in 30 µl. RNA quality was assessed on a bioanalyser (Agilent) according to the manufacturer’s instructions. All samples have a RIN greater than 7. RNA quantity was determined for each sample using a Qubit (Invitrogen, Carlsbad, CA, USA) according to the manufacturer’s instructions.

### Microarray Processing

Microarray experiments were performed according to the manufacturer’s instructions (Affymetrix). Briefly, 125 ng of total RNA were reverse transcribed and labeled using the WT ovation RNA amplification system (Nugen) according to manufacturer’s instructions. The fragmented cRNA was hybridized onto GeneChip® Human Genome U133 Plus 2.0 Array (Affymetrix). Expression data were generated using the Robust Multi-array Average (RMA) method implemented in the Affymetrix package of the Bioconductor microarray analysis environment (http://www.bioconductor.org). The RMA consists of three steps: background adjustment, quantile normalization [15] and probe set summary of the log-normalized data applying a median polishing procedure. To compensate for batch effects, we applied COMBAT [15], [16] during the pre-processing of the data. A moderated paired T-Test was applied to assess the statistical significance of differential expression. We filtered out low-expressed probesets on the arrays for each Differentially Expressed Gene (DEG) analysis by keeping probesets with a median expression of at least 2^6^ for at least one out of the two conditions. Data is available in GEO under the accession number GSE46914.

### Quantitative PCR Analysis

cDNA was synthesized from 100 ng of total RNA using SuperScript® VILO™ system (Invitrogen) according to manufacturer’s instructions. PCR reactions were performed on a LightCycler® 480 instrument using the associated Probe Master Mix according to the manufacturer’s instructions (Roche Molecular Biochemicals, Indianapolis, IN, USA). mRNA expression levels of the housekeeping gene Peptidylpropyl isomerase B (PPIB), encoding for cyclophilin B, and gene expression was investigated using TaqMan Gene Expression Assays (Applied Biosystems, Foster City, CA). The validity of PPIB mRNA levels as reference for target mRNA quantification has been previously demonstrated in human peripheral blood [17]. Data was analyzed using the 2^−ddCt^ method.

### Drug Effects in Cells from Septic Patients

EDTA whole blood was collected from 7 septic patients within 3 days following ICU admission and from 5 healthy volunteers. Septic patients belong to a global study on ICU-induced immune dysfunctions. It has been approved by our Institutional Review Board for ethics (“Comité de Protection des Personnes dans la Recherche Biomédicale de Lyon A, hôpital HotelDieu, Lyon”) which waived the need for informed consent because biomarkers expression was measured on residual blood after completing routine follow-up. The study is registered at French Ministry of Research and Teaching (#DC-2008-509) and is also recorded in our commission for informatics and freedom (“Commission Nationale de l’Informatique et des Libertés”). The purpose of the study was explained to the patients or members of their families. After centrifugation and removal of plasma, 3 ml of blood was diluted 1∶1 with 3 ml of RPMI 1640 medium (Eurobio, Courtaboeuf, France) and then cultured in presence or absence (control group) of 100 ng/ml LPS +/−100 ng/ml rIFN- γ1b (Imukin) for one night at 37°C and 5% CO2 (15 hours). For each condition, supernatants and cell pellets were recovered and stored at –80°C for TNF-α measurement by ELISA and at –20°C for RNA extraction and quantification by qRT-PCR, respectively, as previously described [14].

### Statistical Analysis

Results are expressed as median ± IQR. Statistical analysis was done using the Wilcoxon matched-pairs signed-ranks test for comparison between culture conditions or the Mann Whitney U-test for comparison between septic patients and healthy volunteers. Statistical analyses were performed using Prism Software (version 4.03, GraphPad Software, La Jolla, CA). A p-value <0.05 was considered significant with correction by the number of analyses performed.

## Results

### Transcript Selection in an *in vitro* Model of Tolerant Mononuclear Cells

**Figure 1:**
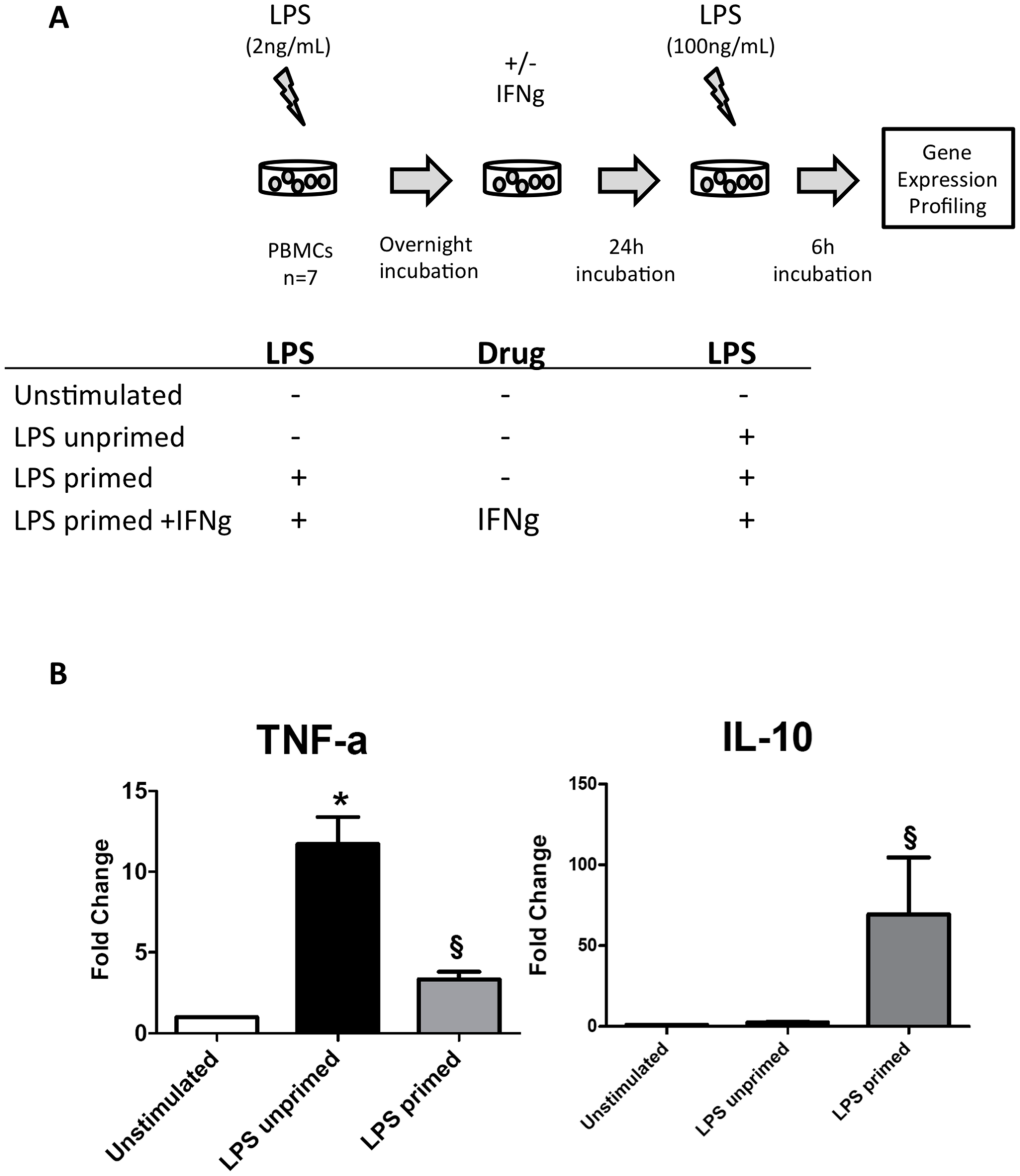
The Endotoxin Tolerance Model. (A). Schematic representation of the endotoxin tolerance model used in the study. (B) The expression of TNF-a and IL-10 was measured by qRT-PCR upon treatment of the cells according to Figure 1A. The Wilcoxon signed rank test was used to test for statistical significance (*p<0.05 Non stimulated vs LPS-unprimed cells; § *p*<0.05 LPS-unprimed cells vs LPS-primed cells).

**Figure 2:**
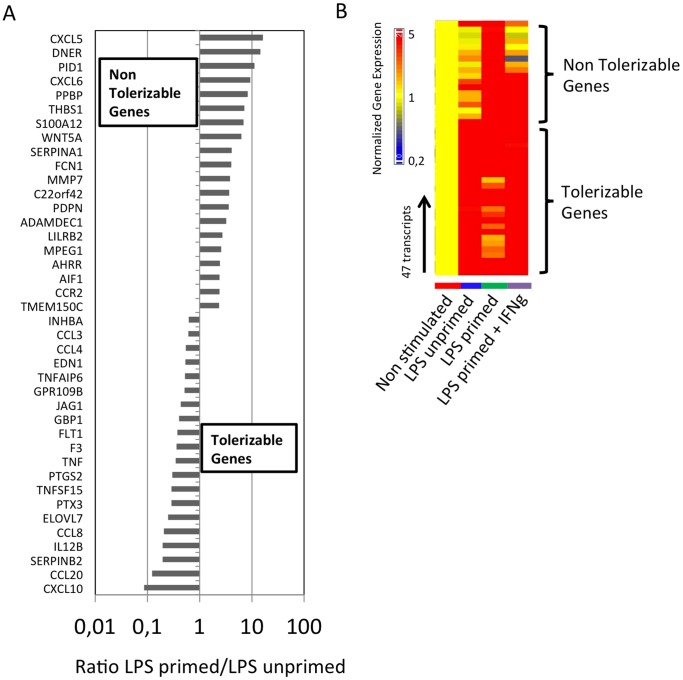
Identification of Biomarkers of Response to IFN-γ. (A). PBMCs from 6 HD were stimulated according to the scheme shown in Figure 1. After the stimulation, RNA was extracted and hybridized to Affymetrix U133 Plus chips. 70 transcripts were found differentially expressed between the « non-stimulated » condition and the « LPS unprimed » condition and 43 between the « LPS unprimed » and « LPS primed » conditions (paired t-test p<0.05, av. FC>2 in at least 4 donors). The ratios “LPS primed”/”LPS unprimed” of the top 20 non tolerizable genes and the top 20 tolerizable genes are represented. (C). Out of the 113 transcripts that were found dysregulated in tolerant monocytes, the expression of 47 transcripts was significantly restablished upon adding rIFN-γ. Those 47 transcripts were arranged by hierarchical clustering to reveal differential expression. Expression values are normalized per gene to the unstimulated condition. Transformed expression levels are indicated by color scale, with red representing relative high expression and blue indicating relative low expression. A list of the genes shown in this figure is available in Table S1.

In an attempt to identify genes differentially expressed in tolerant mononuclear cells, we performed a genome-wide expression analysis using Affymetrix U133 Plus arrays to compare unstimulated PBMCs, PBMCs stimulated once with LPS (LPS-unprimed) and tolerant PBMCs re-stimulated with LPS (LPS-primed) according to Figure 1. Models of ET have been characterized by a reduction of TNF-a production paralleled by an increased IL-10 secretion following two stimulations with LPS. The expression of those two genes was indeed found to follow the expected pattern (Figure 1B) therefore confirming the development of an ET state in our model. Seventy transcripts were found differentially expressed between the « non-stimulated » condition and the « LPS unprimed » condition and 43 between the « LPS unprimed » and « LPS primed » conditions (paired t-test p<0.05, average Fold Change>2 in at least 4 donors). Taken together, we therefore identified 113 transcripts differentially expressed between the three conditions: unstimulated, LPS unprimed and/or LPS primed. A list of those transcripts is available in Table S1. In agreement with the literature [18], [19], the genes could be divided in 2 groups according to their expression pattern. A first group of transcripts can be termed “tolerizable” as their expression is diminished upon re-exposure to LPS and a second group of transcripts can be described as “non tolerizable” since their expression is induced upon reexposure to LPS. The top 20 “tolerizable” and “non tolerizable genes” are shown Figure 2A. Several pro-inflammatory mediators were found to be reduced during endotoxin tolerance including TNF-a, Prostaglandin-endoperoxide synthase (PTGS)-2, Interleukin (IL)-12B, pentraxin (PTX)-3 and Tissue factor 3 (F3). The TIR-domain-containing adapter-inducing interferon-β (TRIF) pathway was also tolerized as several genes known to be induced by this pathway were downregulated in tolerant monocytes like the cytokines CXCL-10 and CCL-5, the IFN-γ inducible genes guanylate binding protein (GBP)-1, GBP-5 and the transcription factor Interferon Regulatory Factor (IRF)-1 (Table S1). Genes induced in tolerant PBMCs included several chemokines like CXCL-1, CXCL-3, CXCL-5, CXCL-6 and CXCL-7. CCR2, the receptor for CCL2, was also upregulated in tolerant PBMCs (Table S1).

Next, we studied the effect of IFN-γ, an immunostimulatory drugs that have been shown to have favorable outcome in sepsis patients in several studies [5], [7], [20]. Out of the 113 transcripts that were found dysregulated in tolerant PBMCs, the expression of 47 transcripts was reestablished upon adding IFN-γ (paired t-test p<0.05) (Table S1). A representation of those genes is shown Figure 2B following hierarchical clustering. The expression of tolerizable genes was upregulated upon addition of IFN-γ. Conversely, the non-tolerizable genes were downregulated by IFN-γ.

**Figure 3:**
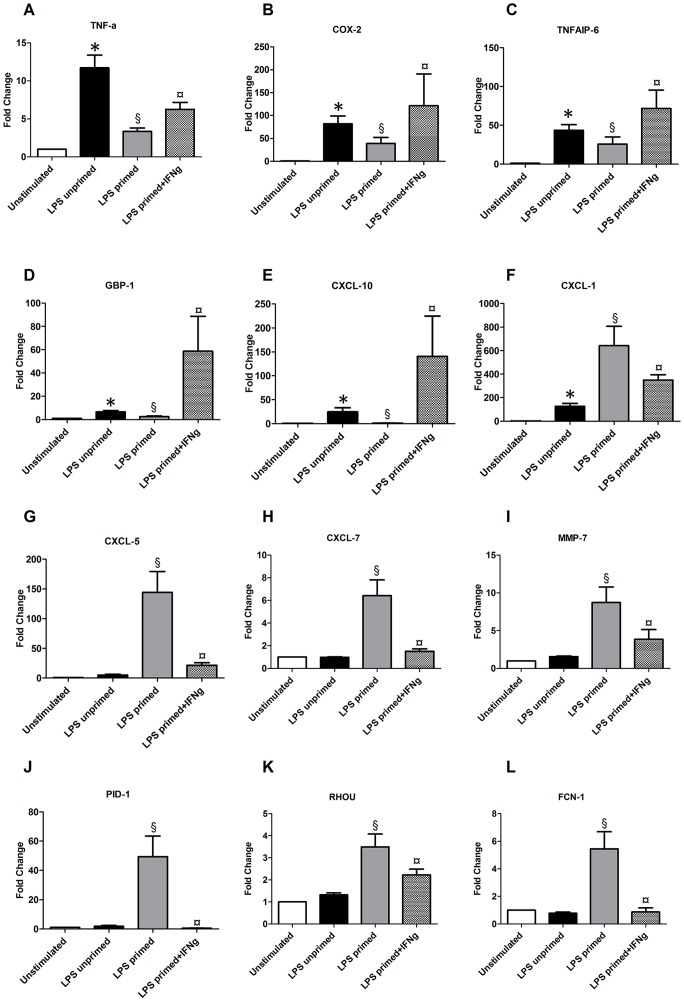
Validation of Twelve Biomarkers of Response to IFN-γ. The expression of 12 genes was validated by qRT-PCR. The Wilcoxon signed rank test was used to test for statistical significance (*p<0.05 Non stimulated vs LPS-unprimed cells; § *p*<0.05 LPS-unprimed cells vs LPS-primed cells; ¤*p*<0.05 LPS-primed cells vs LPS-primed cells+ IFN-γ).

The expression of the top 12 differentially expressed transcripts upon IFN-γ addition (p<0.05 with average |FC|>2 and/or |FC|>2 in at least 3 donors) was validated by qRT-PCR. Out of those 12 genes, five were tolerizable genes and are represented Figure 3A to 3E: two interferon–inducible genes GBP-1 and CXCL-10 and three inflammatory genes TNF-a, COX2 and tumor necrosis factor alpha-induced protein (TNFAIP)-6. Conversely, seven non tolerizable genes are shown Figure 3F to 3L: CXCL-1, CXCL-5, CXCL-7, matrix metalloproteinase (MMP)-7, phosphotyrosine interaction domain containing (PID)-1, ras homolog gene family, member U (RHOU) and Ficolin (FCN)-1.

Altogether, using this *in vitro* approach with healthy volunteers’ cells we identified a list of 47 transcripts whose expression was altered during endotoxin tolerance (tolerizable and non tolerizable genes) and restored by IFN-γ. Among the identified transcripts, a panel of 12 genes will now be validated in septic patients along with the genes IL-10 and HLA-DRA known to be altered in ET and restored by IFNg [14].

### Transcript Validation upon IFN-γ Challenge in Septic Patients Cells *ex vivo*

**Figure 4:**
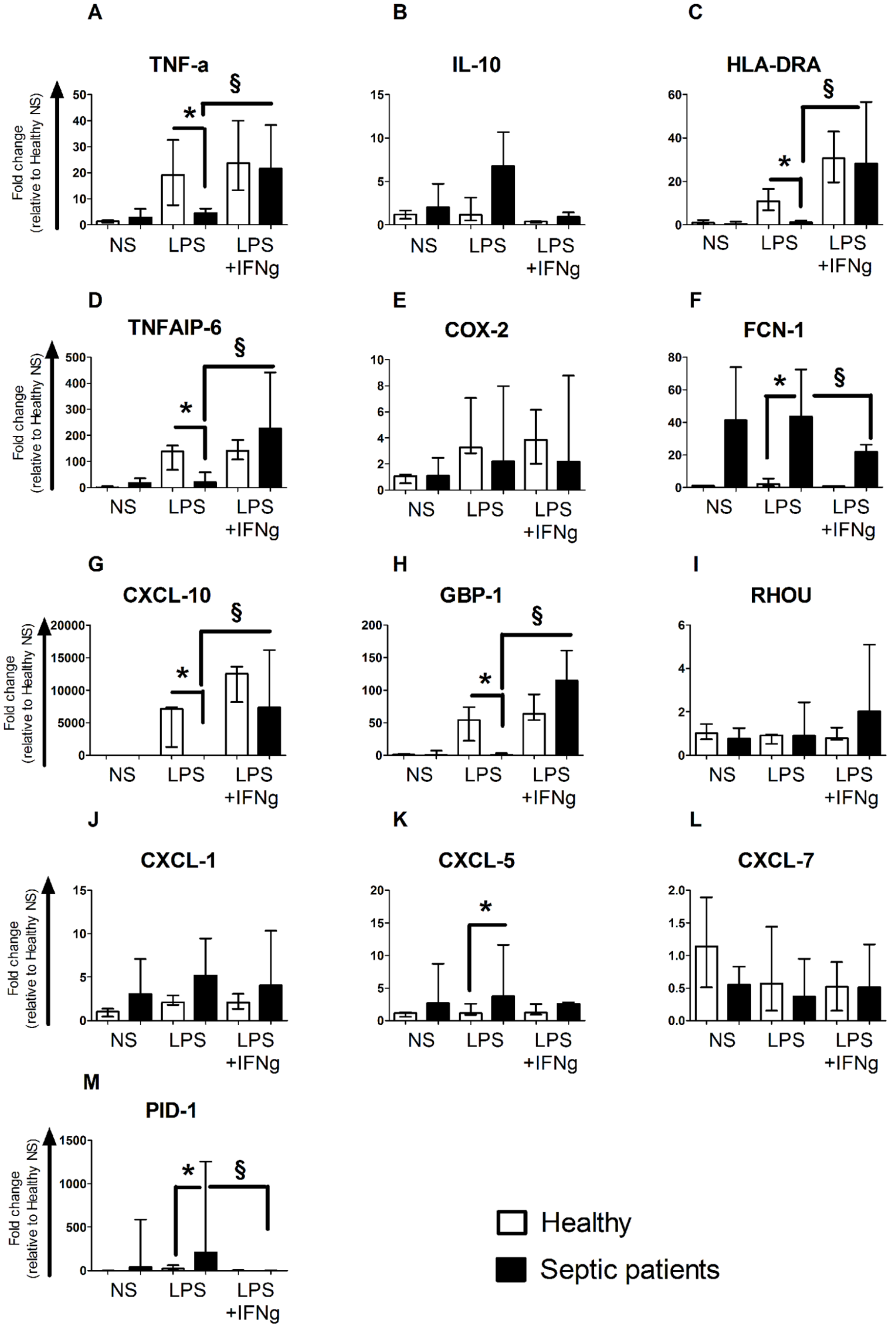
IFN-γ treatment restores the expression of candidates biomarkers in whole blood from septic patients. Whole blood from seven healthy donors (open columns) or septic patients (black columns) was stimulated with LPS (100 ng/ml) ± rIFN-γ1b (100 ng/ml) overnight and the expression of the biomarkers previously identified was measured by qRT-PCR. Data are presented as median ± IQR. Comparison between healthy volunteers and septic patients treated with LPS was performed using the Mann-Whitney U test (* *P*<0.05) whereas evaluation of rIFN-y effect was performed using the Wilcoxon signed rank test (§ *P*<0.05).

We next set out to validate the previously identified transcripts *ex vivo* in whole blood from seven septic patients sampled within 3 days following ICU admission (patients’ details are available in Table 1). Whole blood was activated with LPS +/− IFN-γ and the expression of the 12 transcripts previously identified was monitored by qRT-PCR (Figure 4) Additionally, we also evaluated the expression of two additional genes (IL-10 and HLA-DRA) described as potential markers of immunosuppression [14] but which differential expression in our present microarray data did not meet statistical significance. As expected, the expression of TNF-a was significantly lower in septic patients’ blood than in healthy blood in response to LPS activation. When IFN-γ was added to the culture, the transcription of TNF-a in healthy and septic patients’ blood returned to a comparable level. IL-10 expression was already increased in patients compared to healthy without any stimulation. When LPS was added to the blood, the difference in IL-10 expression increased even more. Adding IFN-γ decreased IL-10 expression in both groups to a lower level than without any stimulation. However, the difference in IL-10 expression between healthy and patients remained statistically significant. Finally, LPS induced the expression of HLA-DRA in healthy but not in septic patients. When IFN-γ was added to the culture, HLA-DRA expression in healthy and patients became comparable.

**Table 1:** Patient’s Characteristics.

Parameters	Patients (n = 7)
Age	65 (62–75)
Number of Males	4
Number of Females	3
Source of infection	Pneumopathy (n = 4)
	CNS infection (n = 2)
	Gastrointestinal infection (n = 1)
Suspected infection	Clinically documented diagnosis (n = 3)
	Microbiologically documented diagnosis (n = 4): Bacilli Gram- (n = 1); Cocci Gram+ (n = 3); Fungi (n = 0)
SOFA	11(10–11.5)
SAPSII	66 (47–81.5)
CHARLSON*	2 (1–2.5)
MACCABE	0 (n = 3) 1 (n = 4)
Treatment	hemisuccinate hydrocortisone (<10 mg/day) (n = 2)
Number of lymphocytes (10^3^/ul)	0.7 (0.4–0.8)
% of HLA-DR+ lymphocytes	59 (47–65)
Mortality (number of survivors)	4

Results are presented as median and interquartile range (Q1–Q3) for continuous variables and as number of cases for categorical variables.

SAPSII, Simplified Acute Physiology Score; SOFA, Sequential Organ Failure Assessment;

* The most common comorbidity observed was myocardial infarcts (2/7 patients) and Moderate or severe liver disease (2/7 patients).

We then studied the expression of the remaining 11 genes previously identified in our *ex vivo* model of endotoxin tolerance. As expected, upon LPS stimulation the expression of CXCL-10, GBP1 and TNFAIP6 was much more reduced in septic patients than in healthy controls. However, by adding IFN-γ the expression level of those three genes reached a level comparable to the one measured in healthy controls. Conversely, the expression of PID1, FCN1 and CXCL-5 was significantly higher in patients than in healthy at the basal level and upon LPS stimulation. Upon IFN-γ addition, those three genes also reached a comparable level than in healthy. The effect of IFN-γ on the expression of COX2, RHOU, CXCL-1 and CXCL-7 was less pronounced. Finally, the expression of MMP7 was too low in whole blood and could not be detected in any of the samples (data not shown).

Overall, we therefore identified 6 novel transcripts (TNFAIP6, FCN1, CXCL-10, GBP1, CXCL-5 and PID1) in addition to TNF, IL-10 and HLA-DRA that are differentially expressed in septic patients’ blood compare to healthy blood upon *ex vivo* LPS stimulation and are restored when IFN-γ is added to the culture.

## Discussion

Over the past 20 years, most of the new therapeutic strategies have focused on using anti-inflammatory drugs and have only had a modest effect on decreasing mortality. Indeed, as our capacity to treat patients during the very first hours of shock has improved, many patients now survive this critical step only to die later in a state of immunosuppression. Consequently, immunostimulatory therapies constitute an innovative strategy that deserves to be assessed for the treatment of sepsis. IFN-γ plays a major role in the biology of the monocyte/macrophage lineage by activating antimicrobial activity, increasing killing of intracellular pathogens, and antigen processing and presentation to lymphocytes through induction of MHC antigens [21]. IFN-γ represents therefore an interesting approach to rescue monocyte functions in sepsis. Indeed, encouraging preliminary studies with IFN-γ have been described in sepsis or trauma patients [7]–[11]. IFN-γ has also been shown to be effective at reversing immunosuppression in several *in vitro* and *in vivo* models of endotoxin tolerance [22]–[25].

However, because of patients’ heterogeneity, we need to be able to identify patients beforehand who would actually benefit from immunostimulatory therapies. The validity of this concept was demonstrated by Docke et al. [7] using monocytic HLA-DR expression as a stratification tool. Furthermore, flow cytometric measurement of HLA-DR has been shown to be a reliable biomarker for the prediction of death and nosocomial infections in sepsis patients [26]–[28] therefore reinforcing the idea that biomarkers of immunosuppression are useful. It remains however difficult to implement flow cytometric assays in large multicentered studies and in routine labs [29]. Gene expression profiling however provides new perspectives as automated tests with standardized methodologies are available. Indeed, several mRNA biomarkers appear to be promising candidates as surrogate markers of sepsis-induced immunosuppression [28], [30]–[33].

Our study highlights the potential of gene expression profiling at identifying potential biomarkers that will help us to recognize patients eligible for immunomodulatory therapies and monitor response to treatment. Using this bench to the beside approach by microarray, we identified a panel of 11 novel markers (4 tolerizable and 7 non-tolerizable) in addition to IL-10, TNFa and HLA-DRA whose expression was altered in tolerant mononuclear cells and reestablished upon adding IFN-γ. We then validated those by qRT-PCR and therefore confirmed our microarray results. Most importantly, upon validation in clinic, our study led to the identification of six novel genes that could be used to follow response to IFN-γ treatment in septic patients; 3 of those being tolerizable (TNFAIP6, GBP1 and CXCL-10) and 3 non-tolerizable (CXCL-5, FCN1 and PID1).

TNFAIP6 is known to be upregulated in response to many proinflammatory mediators (e.g. TNFa) and high levels of TNFAIP6 have been reported in the plasma of patients with bacterial sepsis [34]. Indeed, it appears that TNFAIP6 is a very promising biomarker of response to IFN-γ as its expression was lower in septic patients than in healthy but increased upon *ex vivo* treatment with IFN-γ. Finally, monitoring the expression of both well know IFN-γ inducible genes CXCL-10 and GBP1 appear to be promising as well.

CXCL-5 is a very potent chemoattractant for neutrophils and is an activator of CXCR2. It has been shown that the expression of CXCR2 is reduced on the cell surface of neutrophils and migratory response to the CXC chemokines is markedly suppressed in PMNs from patients with sepsis compared with normal PMNs [35], [36]. It remains to be determined if the effect of IFN-γ on the transcription of CXCL-5 will be beneficial for sepsis patients.

FCN1 is a recognition molecule of the lectin complement pathway. The ficolin-1 gene is polymorphic, but the functional and clinical consequences are unknown. It has recently been shown that homozygosity for the alleles at positions −542 and −144 was significantly associated with fatal outcome in patients with systemic inflammation [37]. Even if the role of Ficolin-1 in sepsis remains to be determined, it is associated with outcome in severe inflammation [38]. PID1 appears to be a promising biomarker as its expression was normalized upon addition of IFN-γ in whole blood culture from septic patients. It has been implicated in glucose homeostasis [39] but its role in the physiopathology of sepsis remains to de determined.

Our study has some limitations. The number of patients used to validate our markers remains small. The population was mainly composed of elderly people who tend to have more comorbidities and receive treatment that could impact the immune defects we observed. We however included patients with a limited number of comorbidities and excluded patients on high corticosteroid dose that are considered immunosuppressive (prednisone dose >10 mg/day). As this preliminary study was designed as a proof of concept study to show that RNA markers could serve as marker of immunosuppression and stratification tool for immunostimulatory treatment, larger studies involving higher number of patients are now warranted.

### Conclusion

Development of immunosuppression early in the course of sepsis is well established and there is a growing interest for immunostimulatory treatments in those patients. However, because of patients’ heterogeneity, we can envision a growing need for biomarkers that will help us stratify patients prior to initiation of those therapies and monitor drug efficacy. In the present study we have identified a panel of 6 genes that are differentially expressed in septic patients upon LPS stimulation and whose expression is restored when adding IFN-γ. The potential value of these newly identified markers along with previously known ones like TNFa, IL-10 and HLA-DRA should now be evaluated in a larger cohort of septic patients. Upon favorable results, we may hypothesize they could serve as a stratification tool prior to immunostimulatory treatment and to monitor drug efficacy.

## Supporting Information

Table S1 List of 113 transcripts differentially expressed between the three conditions: unstimulated, LPS unprimed and/or LPS primed.
(PDF)
