# Correction: Identification of Biomarkers of Response to IFNg during Endotoxin Tolerance: Application to Septic Shock

**DOI:** 10.1371/annotation/5cdfb251-b4ab-4346-b64d-589a3344ef78

**Published:** 2013-12-09

**Authors:** Florence Allantaz-Frager, Fanny Turrel-Davin, Fabienne Venet, Cécile Monnin, Amélie De Saint Jean, Véronique Barbalat, Elisabeth Cerrato, Alexandre Pachot, Alain Lepape, Guillaume Monneret

There is an error in the left column of line 15 in Table 1. "% HLA-DR+ lymphocytes" is incorrect. The correct text for this column is "% HLA-DR+ monocytes." 

