# Supplementary material for: Identification of Biomarkers of Response to IFNg during Endotoxin Tolerance: Application to Septic Shock
Source: PLoS One. 2013 Jul 11;8(7):e68218. doi: 10.1371/journal.pone.0068218 (PMC3708924; doi:10.1371/journal.pone.0068218)
Supplement: Table S1 — List of 113 transcripts differentially expressed between the three conditions: unstimulated, LPS unprimed and/or LPS primed. (PDF) [file pone.0068218.s001.pdf]

Probe Set ID	Gene Symbol	unstimulated vs LPS unprimed					LPS unprimed vs LPS primed					LPS primed vs LPS primed+IFNg					average expression				Gene Title
		mean FC	mean diff. in RD	Raw p-value	FC>2 in	FC<0,5 in	mean FC	mean diff. in RD	Raw p-value	FC>2 in	FC<0,5 in	mean FC	mean diff. in RD	Raw p-value	FC>2 in	FC<0,5 in	unst.	LPS unprimed	LPS primed	LPS primed + IFNg	
1554997_a_at	PTGS2*	23,19	300,37	1,10E-02	5	0	0,55	-146,30	4,10E-02	0	4	1,72	69,15	4,74E-02	3	0	75,64	333,11	174,19	227,19	prostaglandin-endoperoxide synthase 2
1555990_at	C22orf42	1,06	-3,70	ns	0	0	3,18	82,24	1,89E-02	4	0	1,54	-9,71	ns	1	1	43,49	40,32	110,59	122,11	chromosome 22 open reading frame 42
1555991_s_at	C22orf42	1,41	1,97	ns	0	0	3,54	73,39	9,88E-03	4	0	1,21	-18,45	ns	1	1	31,52	33,21	97,53	97,37	chromosome 22 open reading frame 42
201107_s_at	THBS1	0,85	-20,14	ns	0	1	6,22	78,67	0,04376	5	0	0,95	-30,03	ns	1	2	60,36	43,10	167,31	107,81	thrombospondin 1
201108_s_at	THBS1	0,87	-44,10	5,70E-02	0	0	7,34	236,09	3,43E-02	5	1	0,74	-127,57	ns	1	4	184,44	146,64	519,35	303,97	thrombospondin 1
201110_s_at	THBS1	1,14	-10,76	ns	1	0	7,42	64,31	3,05E-02	5	0	0,49	-58,83	1,17E-02	0	6	53,26	44,03	151,16	61,12	thrombospondin 1
201631_s_at	IER3	2,44	3152,89	6,21E-03	4	0	1,00	40,08	ns	0	0	0,96	621,09	ns	0	1	3456,61	6159,09	6156,33	5875,84	immediate early response 3
201668_x_at	MARCKS	3,00	176,04	7,13E-03	4	0	1,04	16,55	ns	0	0	0,95	15,23	ns	0	0	147,32	298,21	307,72	283,96	myristoylated alanine-rich protein kinase C substrate
201669_s_at	MARCKS	3,81	793,20	1,45E-02	4	0	1,08	-10,39	ns	0	0	0,81	-66,74	6,02E-03	0	0	577,00	1256,88	1306,04	1056,03	myristoylated alanine-rich protein kinase C substrate
201670_s_at	MARCKS	2,81	2020,28	6,77E-03	4	0	1,00	-98,46	ns	0	0	0,93	213,14	ns	0	0	1742,54	3474,21	3441,98	3176,82	myristoylated alanine-rich protein kinase C substrate
201887_at	IL13RA1	0,78	-51,64	2,16E-02	0	0	1,93	122,75	7,30E-05	4	0	1,01	10,23	ns	0	0	206,86	162,59	307,68	309,26	interleukin 13 receptor, alpha 1
201963_at	ACSL1	2,03	595,83	1,52E-02	4	0	0,83	-217,46	ns	0	1	1,10	169,66	ns	0	0	749,58	1260,29	973,56	1020,10	acyl-CoA synthetase long-chain family member 1
202269_x_at	GBP1*	2,88	1929,30	2,36E-02	4	0	0,72	-433,82	8,98E-02	1	3	5,95	3822,15	7,24E-03	5	0	1481,70	3135,38	1652,32	6846,33	guanylate binding protein 1, interferon-inducible, 67kDa
202270_at	GBP1	3,82	107,51	3,04E-02	4	0	1,02	-7,36	ns	1	4	9,16	220,78	1,62E-02	6	1	57,89	150,04	84,69	410,54	guanylate binding protein 1, interferon-inducible, 67kDa
202833_s_at	SERPINA1	1,27	10,94	ns	1	0	4,25	270,52	2,34E-03	5	0	1,39	94,75	2,04E-02	2	0	124,60	133,98	454,38	592,99	serpin peptidase inhibitor, clade A (alpha-1 antiproteinase, antitrypsin), member 1
202838_at	FUCA1	0,65	-112,95	2,11E-02	0	2	1,96	108,43	2,51E-02	4	0	0,94	-20,50	ns	0	0	250,03	153,22	295,93	265,31	fucosidase, alpha-L- 1, tissue
202859_x_at	IL8	2,43	2439,43	7,68E-03	4	0	0,93	-389,86	9,16E-02	0	0	0,87	204,85	1,10E-03	0	0	2595,02	4685,96	4326,88	3772,06	interleukin 8
204103_at	CCL4	3,07	2187,90	1,36E-02	4	0	0,66	-1033,09	3,04E-02	0	3	1,15	581,52	ns	0	0	1783,70	3659,05	2208,16	2404,35	chemokine (C-C motif) ligand 4
204259_at	MMP7*	1,31	49,88	4,33E-02	0	0	3,27	401,66	8,30E-03	4	0	0,67	-152,16	3,48E-02	0	3	279,76	322,52	803,76	514,47	matrix metallopeptidase 7
204363_at	F3	2,29	353,24	7,66E-03	4	0	0,38	-366,41	2,49E-03	0	6	1,27	113,22	ns	0	1	406,28	709,06	215,82	250,79	coagulation factor III (thromboplastin, tissue factor)
204470_at	CXCL1*	60,28	1599,10	9,88E-03	6	0	2,79	1627,96	8,43E-03	3	0	0,72	-579,87	2,73E-04	0	0	505,18	1875,83	4272,31	3066,28	chemokine (C-X-C motif) ligand 1
204533_at	CXCL10*	17,37	543,92	3,40E-02	4	0	1,18	-310,25	3,70E-02	1	6	62,29	742,30	1,22E-02	5	1	82,48	548,70	71,19	1021,96	chemokine (C-X-C motif) ligand 10
204614_at	SERPINB2	31,85	534,85	9,35E-03	5	0	0,44	-323,89	1,73E-02	0	4	1,27	38,60	ns	2	1	121,59	580,03	220,98	216,69	serpin peptidase inhibitor, clade B (ovalbumin), member 2
204748_at	PTGS2	25,81	749,67	5,82E-03	6	0	0,49	-384,46	2,12E-02	0	4	1,83	180,20	ns	3	0	167,71	810,29	371,09	512,68	prostaglandin-endoperoxide synthase 2
204879_at	PDPN	1,28	12,06	ns	0	0	2,23	61,32	5,50E-03	5	0	0,87	-7,90	ns	0	0	59,42	69,75	137,97	115,71	podoplanin
204926_at	INHBA	2,58	55,11	5,12E-03	4	0	0,72	-17,93	6,20E-02	0	2	1,82	29,83	4,11E-02	2	0	47,35	94,59	64,61	102,09	inhibin, beta A
205067_at	IL1B	36,60	3674,62	7,16E-03	6	0	0,79	-625,49	4,79E-02	0	1	0,96	277,35	ns	1	0	1161,94	4311,61	3422,28	3101,28	interleukin 1, beta
205114_s_at	CCL3	2,50	1357,93	1,45E-02	4	0	0,69	-593,19	2,27E-02	0	2	1,01	307,42	ns	0	0	1412,33	2576,27	1653,07	1614,56	chemokine (C-C motif) ligand 3
205207_at	IL6	26,30	548,07	1,17E-02	5	0	1,93	176,50	ns	2	2	1,20	-22,86	ns	1	1	151,99	621,76	928,64	857,64	interleukin 6
205220_at	GPR109B	4,70	123,28	3,59E-02	4	0	0,83	45,40	ns	1	3	4,41	241,22	1,21E-02	5	0	103,46	209,14	161,50	512,46	G protein-coupled receptor 109B
205237_at	FCN1*	0,85	-24,89	ns	0	1	4,15	154,93	1,48E-02	5	0	0,55	-153,47	2,02E-02	2	5	87,26	65,93	266,79	71,80	ficolin (collagen/fibrinogen domain containing) 1
205476_at	CCL20	21,26	454,54	8,24E-03	5	0	0,20	-370,18	4,55E-04	0	6	1,04	59,86	ns	0	2	116,92	506,53	76,65	68,79	chemokine (C-C motif) ligand 20
205599_at	TRAF1	2,52	187,26	1,66E-02	4	0	0,97	20,14	ns	0	0	1,09	39,56	ns	1	0	207,97	368,48	341,19	347,82	TNF receptor-associated factor 1
205767_at	EREG	2,03	37,06	5,40E-02	2	0	1,84	78,55	1,25E-02	4	0	0,47	-95,10	5,26E-04	0	4	100,62	132,39	234,29	104,65	epiregulin
205863_at	S100A12	1,20	8,91	ns	0	0	8,23	195,65	1,87E-02	5	0	0,78	-102,40	ns	1	3	72,91	80,55	344,25	206,37	S100 calcium binding protein A12
205990_s_at	WNT5A	2,37	5,96	ns	2	0	5,22	55,77	3,86E-03	6	0	1,34	12,46	ns	1	0	20,09	25,20	94,50	117,44	wingless-type MMTV integration site family, member 5A
206025_s_at	TNFAIP6*	14,56	1477,68	1,10E-02	4	0	0,61	-305,25	2,33E-02	0	4	2,38	848,82	1,61E-02	3	0	688,59	1955,17	1156,53	2145,00	tumor necrosis factor, alpha-induced protein 6
206026_s_at	TNFAIP6	13,57	1611,02	8,15E-03	5	0	0,67	-376,69	3,35E-02	0	3	1,68	530,45	ns	2	0	659,79	2040,66	1275,96	1894,58	tumor necrosis factor, alpha-induced protein 6
206134_at	ADAMDEC1	1,40	24,19	4,90E-02	0	0	3,78	212,31	6,43E-03	5	0	1,32	52,63	ns	0	0	101,89	122,63	376,88	463,30	ADAM-like, decysin 1
206157_at	PTX3	4,38	206,58	1,36E-02	5	0	0,66	-168,02	7,05E-02	0	5	0,70	-39,32	ns	1	4	85,83	262,90	108,15	23,86	pentraxin 3, long
206336_at	CXCL6	1,68	2,39	ns	1	0	9,32	42,80	4,54E-03	6	0	0,51	-31,93	1,37E-02	0	5	11,11	13,15	69,37	26,21	chemokine (C-X-C motif) ligand 6
206360_s_at	SOCS3	3,44	58,41	3,70E-03	5	0	1,17	16,02	ns	0	1	1,24	17,83	ns	1	1	40,92	90,98	106,32	116,69	suppressor of cytokine signaling 3
206380_s_at	CFP	1,13	2,73	ns	0	0	2,19	41,17	2,07E-02	4	0	0,73	-26,50	ns	0	2	46,30	48,64	101,80	61,30	complement factor properdin
206414_s_at	ASAP2	3,29	62,30	9,92E-03	4	0	0,84	-6,36	ns	0	1	1,01	11,26	ns	0	0	65,32	118,72	92,30	88,58	ArfGAP with SH3 domain, ankyrin repeat and PH domain 2
206978_at	CCR2	0,71	-21,33	4,41E-02	0	1	3,05	51,50	3,01E-02	5	0	0,72	-30,89	ns	0	2	68,34	50,05	124,58	78,50	chemokine (C-C motif) receptor 2
207085_x_at	CSF2RA	0,94	-4,16	ns	0	0	2,19	42,31	5,96E-03	4	0	0,81	-17,68	ns	0	1	46,98	43,41	93,64	66,53	colony stimulating factor 2 receptor, alpha, low-affinity
207113_s_at	TNF*	7,23	678,57	8,07E-03	4	0	0,50	-329,14	1,25E-02	0	6	2,08	326,38	3,11E-02	2	0	389,46	971,09	351,90	629,53	tumor necrosis factor
207275_s_at	ACSL1	2,24	529,42	1,67E-02	4	0	0,71	-220,75	4,58E-02	0	3	1,41	277,99	ns	1	0	630,01	1083,80	712,14	924,08	acyl-CoA synthetase long-chain family member 1
207277_at	CD209	0,98	-3,39	ns	0	0	2,06	31,67	3,67E-02	4	0	1,16	2,12	ns	1	0	49,37	46,47	82,65	87,99	CD209 molecule
207533_at	CCL1	8,35	221,90	9,85E-03	5	0	0,83	18,98	ns	0	2	1,25	177,24	ns	1	0	242,49	432,69	226,35	319,09	chemokine (C-C motif) ligand 1
207850_at	CXCL3	11,65	651,13	9,95E-03	4	0	1,12	78,76	ns	1	0	0,84	-46,49	3,94E-02	0	0	333,74	891,85	979,60	775,82	chemokine (C-X-C motif) ligand 3
207901_at	IL12B	16,80	74,77	1,34E-02	4	0	0,43	-40,22	8,47E-03	0	6	4,63	31,58	ns	3	0	35,76	99,85	21,50	54,68	interleukin 12B
208075_s_at	CCL7	2,45	64,39	8,26E-03	4	0	0,84	89,46	ns	0	1	5,13	175,50	6,90E-03	4	0	72,19	127,39	107,88	437,46	chemokine (C-C motif) ligand 7
209099_x_at	JAG1	2,22	268,33	1,61E-02	4	0	0,48	-267,66	1,53E-02	0	5	1,80	144,40	ns	3	0	375,51	605,51	197,92	289,11	jagged 1
209267_s_at	SLC39A8	3,26	477,18	1,69E-02	4	0	1,07	14,54	ns	1	1	1,13	106,65	ns	1	0	429,10	838,11	818,62	853,93	solute carrier family 39 (zinc transporter), member 8
209774_x_at	CXCL2	7,25	1900,88	7,59E-03	4	0	0,76	-496,67	2,18E-02	0	0	0,81	-18,46	1,28E-02	0	0	936,38	2565,71	1989,52	1564,62	chemokine (C-X-C motif) ligand 2
209901_x_at	AIF1	0,79	-143,44	6,26E-02	0	0	2,61	688,33	6,17E-04	5	0	1,17	78,61	ns	0	0	637,84	514,89	1278,83	1431,84	allograft inflammatory factor 1
210029_at	IDO1	2,93	186,72	2,70E-02	4	0	2,12	932,95	ns	4	0	15,14	3242,35	3,49E-03	5	0	161,49	321,53	579,81	5228,18	indoleamine 2,3-dioxygenase 1
210118_s_at	IL1A	9,97	203,98	8,57E-03	5	0	0,90	-22,33	ns	0	1	1,42	60,57	1,16E-02	1	0	84,31	259,15	225,55	295,51	interleukin 1, alpha
210146_x_at	LILRB2	0,94	-22,80	ns	0	0	3,00	356,08	5,17E-03	5	0	1,16	-11,67	ns	1	0	268,24	248,70	650,71	667,56	leukocyte immunoglobulin-like receptor, subfamily B , member 2
210340_s_at	CSF2RA	0,96	-7,01	ns	0	0	2,06	61,36	7,02E-03	4	0	0,75	-27,80	2,02E-02	0	0	83,59	77,58	155,06	107,88	colony stimulating factor 2 receptor, alpha, low-affinity (granulocyte-macrophage)
210511_s_at	INHBA	2,69	570,55	1,00E-02	4	0	0,81	-78,95	ns	0	2	1,92	476,43	1,18E-02	2	0	551,97	1041,02	780,63	1317,70	inhibin, beta A
210660_at	LILRA1	0,87	-21,22	ns	0	0	1,93	84,13	7,61E-03	4	0	0,78	-34,48	ns	0	1	142,20	124,02	224,18	161,03	leukocyte immunoglobulin-like receptor, subfamily A, member 1
211286_x_at	CSF2RA	0,91	-7,94	ns	0	0	1,84	45,26	1,06E-02	4	0	0,91	-10,27	ns	0	0	72,12	65,32	118,48	97,63	colony stimulating factor 2 receptor, alpha, low-affinity
211429_s_at	SERPINA1	1,28	45,94	ns	1	0	4,57	728,29	2,03E-03	5	0	1,37	189,04	3,48E-02	1	0	274,61	313,99	1160,07	1465,03	serpin peptidase inhibitor, clade A , member 1
211506_s_at	IL8	3,89	2708,68	1,11E-02	4	0	0,88	-207,91	7,59E-02	0	0	1,03	469,31	ns	0	0	2063,72	4385,44	3902,52	3897,91	interleukin 8
213002_at	MARCKS	2,45	3021,04	8,46E-03	4	0	1,02	92,73	ns	0	0	0,94	350,14	ns	0	0	3163,69	5753,16	5867,21	5449,98	myristoylated alanine-rich protein kinase C substrate
213095_x_at	AIF1	0,83	-121,44	8,56E-02	0	0	2,51	636,61	5,72E-04	5	0	1,09	31,33	ns	0	0	626,24	522,15	1249,50	1314,82	allograft inflammatory factor 1
213201_s_at	TNNT1	0,91	-3,28	ns	0	0	2,74	24,86	2,40E-02	5	0	0,59	-21,22	7,93E-03	0	3	27,06	24,25	58,76	28,86	troponin T type 1 (skeletal, slow)
213524_s_at	G0S2	3,75	864,98	6,92E-03	4	0	0,99	-8,05	ns	0	0	0,67	7,69	3,22E-02	0	2	845,32	1586,73	1485,60	919,00	G0/G1switch 2
214022_s_at	IFITM1	1,89	116,01	3,28E-03	4	0	0,89	-4,38	ns	0	1	2,03	149,02	1,44E-02	4	1	154,59	254,02	214,39	391,69	interferon induced transmembrane protein 1 (9-27)
214038_at	CCL8	25,03	215,51	1,77E-02	4	0	1,47	511,21	ns	1	5	77,85	1883,75	6,20E-03	5	1	42,34	227,06	190,78	3013,77	chemokine (C-C motif) ligand 8
214146_s_at	CXCL7*	0,95	-2,42	ns	0	0	7,45	518,34	2,54E-02	6	1	0,60	-470,33	2,72E-02	2	5	266,04	263,97	899,80	276,52	chemokine (C-X-C motif) ligand 7
214974_x_at	CXCL5*	3,67	148,35	3,10E-02	3	0	10,84	754,45	4,66E-03	6	0	0,40	-590,54	3,93E-03	0	6	177,74	304,90	1431,27	481,59	chemokine (C-X-C motif) ligand 5
215051_x_at	AIF1	0,84	-143,86	9,04E-02	0	0	2,39	748,74	1,15E-03	5	0	1,04	7,66	ns	0	1	813,73	690,43	1565,73	1581,04	allograft inflammatory factor 1
215078_at	SOD2	2,63	132,70	1,56E-02	4	0	1,01	-22,93	ns	1	1	0,97	0,27	ns	0	0	129,59	243,33	230,63	206,74	superoxide dismutase 2, mitochondrial
215101_s_at	CXCL5	2,46	97,11	3,04E-03	2	0	17,01	1278,85	3,04E-03	5	0	0,42	-1023,00	8,12E-03	0	5	185,19	268,43	1862,46	526,94	chemokine (C-X-C motif) ligand 5
215223_s_at	SOD2	2,43	1811,52	1,17E-02	4	0	0,80	-331,73	7,87E-02	0	1	1,41	920,16	ns	1	1	1912,29	3465,02	2677,53	3524,94	superoxide dismutase 2, mitochondrial
216268_s_at	JAG1	2,33	268,57	1,53E-02	4	0	0,51	-256,21	2,07E-02	0	5	1,73	133,36	ns	2	1	337,50	567,70	176,27	244,64	jagged 1
216504_s_at	SLC39A8	3,38	302,75	2,97E-02	4	0	1,12	14,39	ns	1	1	0,98	-26,25	ns	1	0	269,57	529,07	529,07	458,69	solute carrier family 39 (zinc transporter), member 8
216598_s_at	CCL2	5,25	1120,64	8,70E-03	5	0	1,13	345,41	ns	1	0	2,81	1735,22	3,94E-03	4	0	613,52	1574,07	1670,15	4048,77	chemokine (C-C motif) ligand 2
216841_s_at	SOD2	2,29	1259,44	6,68E-03	4	0	0,83	-397,09	ns	0	1	1,08	313,24	ns	0	0	1347,76	2427,28	1923,96	2009,42	superoxide dismutase 2, mitochondrial
218995_s_at	EDN1	2,39	69,24	1,14E-02	4	0	0,57	-50,29	2,10E-02	0	5	1,05	24,19	ns	0	1	94,63	153,97	73,55	69,55	endothelin 1
219093_at	PID1*	1,44	23,43	ns	2	0	12,29	300,22	2,65E-02	6	1	0,61	-333,27	8,02E-03	1	5	69,59	89,67	509,99	45,02	phosphotyrosine interaction domain containing 1
220655_at	TNIP3	5,30	470,34	8,90E-03	4	0	1,51	137,73	ns	2	0	0,62	-167,47	1,22E-03	0	1	263,25	666,40	940,19	578,92	TNFAIP3 interacting protein 3
221085_at	TNFSF15	4,51	77,20	7,43E-03	4	0	0,35	-68,36	4,94E-03	0	6	1,06	15,00	ns	1	2	46,96	113,13	23,64	22,36	tumor necrosis factor (ligand) superfamily, member 15
221477_s_at	SOD2	2,54	542,42	1,40E-02	4	0	0,84	-100,24	ns	0	1	1,17	171,86	ns	0	0	569,59	1034,52	842,85	943,43	superoxide dismutase 2, mitochondrial
221898_at	PDPN	1,43	28,66	3,69E-02	1	0	3,41	117,91	8,79E-03	5	0	0,75	-28,07	ns	0	1	77,17	101,74	241,07	175,36	podoplanin
222802_at	EDN1	3,40	45,68	9,68E-03	4	0	0,54	-31,73	1,54E-02	0	5	0,88	8,22	ns	0	1	46,80	85,95	38,29	29,88	endothelin 1
223168_at	RHOU*	1,09	3,48	ns	0	0	2,13	133,23	1,19E-02	4	0	0,86	-49,40	1,61E-02	0	1	141,66	144,64	300,83	219,27	ras homolog gene family, member U
223217_s_at	NFKBIZ	2,96	325,61	0,010775	4	0	0,76	-121,84	6,92E-02	0	2	1,01	43,55	ns	0	0	287,61	566,70	435,17	401,02	nuclear factor of kappa light polypeptide gene enhancer in B-cells inhibitor, zeta
223278_at	GJB2	1,74	84,28	1,36E-02	2	0	1,87	175,54	1,49E-02	4	0	1,06	-30,55	ns	0	0	154,64	226,88	414,29	406,01	gap junction protein, beta 2, 26kDa
223484_at	C15orf48	2,76	930,26	1,06E-02	4	0	0,78	-76,84	8,61E-02	0	1	1,25	408,91	ns	1	0	999,98	1797,34	1307,80	1589,52	chromosome 15 open reading frame 48
223834_at	CD274	2,13	333,98	0,01285	4	0	0,93	31,37	ns	0	1	2,45	525,20	1,19E-02	5	0	401,44	687,71	588,83	1257,61	CD274 molecule
225897_at	MARCKS	4,93	354,18	1,60E-02	4	0	1,06	-3,00	ns	0	0	0,86	-7,06	4,54E-02	1	0	221,58	525,16	549,07	450,19	myristoylated alanine-rich protein kinase C substrate
226281_at	DNER	1,26	2,76	ns	1	0	20,67	78,96	2,71E-02	6	1	0,79	-79,23	2,65E-02	1	5	11,86	14,23	120,35	14,34	delta/notch-like EGF repeat containing
226497_s_at	FLT1	2,42	52,23	5,11E-03	4	0	0,40	-54,22	1,94E-03	0	4	0,58	-1,76	ns	1	6	49,31	94,07	36,35	15,61	fms-related tyrosine kinase 1
226560_at	---	3,99	135,31	2,06E-02	4	0	0,87	-15,40	ns	0	0	0,79	-6,96	ns	0	2	111,09	227,07	195,64	136,69	---
226818_at	MPEG1	1,04	-1,20	ns	0	0	2,62	135,40	1,96E-02	5	0	0,94	-2,84	9,78E-03	0	0	156,39	155,36	349,36	330,49	macrophage expressed 1
226841_at	MPEG1	0,92	-34,89	ns	0	0	2,97	335,68	9,82E-03	4	0	1,06	13,59	ns	0	0	263,90	233,99	661,64	667,04	macrophage expressed 1
227180_at	ELOVL7	7,64	58,49	1,09E-02	4	0	0,40	-40,29	7,39E-03	0	5	1,45	14,73	ns	1	0	29,25	79,39	24,31	27,83	ELOVL family member 7, elongation of long chain fatty acids
227697_at	SOCS3	4,04	1171,54	5,98E-03	4	0	1,04	-17,49	ns	0	1	1,01	49,35	ns	0	0	735,30	1739,47	1743,98	1607,98	suppressor of cytokine signaling 3
229242_at	---	5,56	684,32	4,67E-03	5	0	0,31	-577,25	6,03E-03	0	6	1,02	138,05	ns	0	2	358,72	945,28	186,06	178,42	---
229354_at	AHRR	0,78	-18,49	ns	0	0	2,57	39,91	2,03E-02	5	0	0,85	-10,49	ns	1	0	59,34	43,48	93,22	77,52	aryl-hydrocarbon receptor repressor
229623_at	TMEM150C	1,29	5,31	ns	1	0	2,40	21,32	2,27E-02	4	0	0,60	-14,11	9,58E-03	0	4	28,19	32,75	68,03	36,62	Transmembrane protein 150C
230741_at	---	2,15	415,25	9,31E-03	4	0	0,65	-232,33	2,75E-02	0	4	1,81	302,84	2,58E-02	2	1	481,48	837,40	458,92	765,48	---
231227_at	WNT5A	1,60	1,83	ns	2	0	5,84	50,48	3,77E-04	7	0	1,28	10,67	4,30E-02	1	0	15,14	16,71	76,55	95,67	Wingless-type MMTV integration site family, member 5A
231577_s_at	GBP1	2,98	1173,58	2,53E-02	4	0	0,75	-278,93	ns	1	3	6,17	2438,48	9,01E-03	5	0	881,95	1887,87	1030,63	4291,96	guanylate binding protein 1, interferon-inducible, 67kDa
232504_at	LOC285628	2,91	50,79	1,30E-02	4	0	0,87	-20,85	ns	0	1	0,76	-2,42	ns	1	1	41,25	84,78	67,20	47,92	hypothetical protein LOC285628
237867_s_at	PID1	1,10	3,88	ns	0	0	3,78	51,10	3,24E-02	5	1	0,61	-48,69	3,77E-02	1	4	27,62	30,94	96,54	29,82	phosphotyrosine interaction domain containing 1
238725_at	IRF1	2,39	187,41	2,12E-02	4	0	0,81	-41,73	ns	1	2	3,46	344,06	7,77E-03	5	1	183,05	343,69	227,71	652,11	interferon regulatory factor 1
238727_at	---	3,65	50,56	2,43E-02	4	0	1,12	5,03	ns	0	0	0,74	-11,28	3,74E-02	1	1	45,20	88,54	95,38	64,98	---
39402_at	IL1B	31,15	3776,35	7,01E-03	6	0	0,79	-715,57	5,99E-02	0	1	0,92	195,97	ns	0	0	1161,17	4398,04	3474,55	3045,83	interleukin 1, beta
